# Occupational exposure to pesticides and endometrial cancer in the Screenwide case-control study

**DOI:** 10.1186/s12940-023-01028-0

**Published:** 2023-11-02

**Authors:** Arnau Peñalver-Piñol, Yolanda Benavente, Jon Frias-Gomez, Juan Alguacil, Miguel Santibañez, Manuel Contreras-Llanes, Paula Peremiquel-Trillas, Marta López-Querol, Sonia Paytubi, Beatriz Pelegrina, Irene Onieva, José Manuel Martínez, Sergi Fernandez-Gonzalez, Javier de Francisco, Víctor Caño, Joan Brunet, Marta Pineda, Jordi Ponce, Xavier Matias-Guiu, Francesc Xavier Bosch, Silvia de Sanjosé, Laia Alemany, Laura Costas

**Affiliations:** 1 Unit of Molecular Epidemiology and Genetics in Infections and Cancer, Cancer Epidemiology Research Programme, Catalan Institute of Oncology. IDIBELL, Av Gran Vía 199-203, L’Hospitalet de Llobregat, Barcelona, 08908 Spain; 2 Servei de Medicina Preventiva i Epidemiologia, Vall d’Hebron Hospital Universitari, Vall d’Hebron Barcelona Hospital Campus, Passeig Vall d’Hebron 119-129, Barcelona, 08035 Spain; 3 Consortium for Biomedical Research in Epidemiology and Public Health - CIBERESP. Carlos III In-stitute of Health, Av. De Monforte de Lemos 5, Madrid, 28029 Spain; 4 Faculty of Medicine, University of Barcelona, Barcelona, Spain; 5 Dept. of Sociology, Social Work and Public Health, Research Group “Preventive Medicine and Public Health”, Center for Research in Health and Environment (CYSMA), Huelva, Spain; 6 University of Cantabria - IDIVAL, Santander, Spain; 7 Dept. of Integrated Sciences, Center for Research in Natural Resources, Health and Environment (RENSMA), Faculty of Experimental Sciences, Research Group “Radiation Physics and Environment” (FRYMA), Campus El Carmen s/n, Huelva, 21007 Spain; 8 Department of Gynecology, Hospital Universitari de Bellvitge, IDIBELL. Hospitalet de Llobregat, Barcelona, Spain; 9 Department of Anesthesiology, Hospital Universitari de Bellvitge, IDIBELL. Hospitalet de Llobregat, Barcelona, Spain; 10 Consortium for Biomedical Research in Cancer – CIBERONC. Carlos III Institute of Health, Av. De Monforte de Lemos 5, Madrid, 28029 Spain; 11 Hereditary Cancer Program, IDIBELL, ONCOBELL Program, Catalan Institute of Oncology, L’Hospitalet, Barcelona, Spain; 12 Medical Oncology Department, Catalan Institute of Oncology, Doctor Josep Trueta Girona University Hospital, Girona, Spain; 13 Hereditary Cancer Program, Catalan Institute of Oncology, IDIBGI, Girona, Spain; 14 Department of Pathology, Hospital Universitari de Bellvitge, IDIBELL. Hospitalet de Llobregat, Bar-celona, Spain; 15 Universitat Oberta de Catalunya, Barcelona, Spain; 16 ISGlobal, Barcelona, Spain

**Keywords:** Pesticides, Job-exposure matrix, Endometrial cancer, Occupational exposure

## Abstract

**Background:**

Endometrial cancer is the most common gynaecological tumour in developed countries and disease burden is expected to increase over the years. Identifying modifiable risk factors may help developing strategies to reduce the expected increasing incidence of these neoplasms.

**Objective:**

This study evaluates the association between occupational exposure to pesticides and endometrial cancer using data from a recent case-control study in Spain.

**Methods:**

The analyses included data from 174 consecutive incident endometrial cancer cases and 216 hospital controls frequency-matched by age. Data were collected through structured epidemiological questionnaires and exposure to pesticides was assessed using a Spanish job-exposure matrix (MatEmESp).

**Results:**

Overall, 12% of controls and 18% of cases were occupationally exposed to pesticides. We observed a positive association between occupational exposure to pesticides and endometrial cancer (OR = 2.08; 95% CI = 1.13–3.88 compared to non-exposed). In general, exposures that occurred farther in the past were significantly associated with endometrial cancer. Exposure to insecticides, fungicides and herbicides were positively associated with endometrial cancer (OR = 2.08; 95% CI = 1.13–3.88, OR = 4.40; 95% CI = 1.65–13.33, and OR = 5.25; 95% CI = 1.84–17.67, respectively). The agricultural, poultry and livestock activities scenario was associated with endometrial cancer (OR = 4.16; 95% CI = 1.59–12.32), while the cleaning exposure scenario was not (OR = 1.22; 95% CI = 0.55–2.67).

**Conclusions:**

Assessment of occupational exposure to pesticides assessed using a Spanish job-exposure matrix revealed a positive association with endometrial cancer. The elucidation of the role of pesticide compounds on endometrial cancer should shed a light on the aetiology of this tumour.

**Supplementary Information:**

The online version contains supplementary material available at 10.1186/s12940-023-01028-0.

## Introduction

Endometrial cancer is the most common gynaecological tumour in developed countries and disease burden is expected to increase over the years [1]. Among women, endometrial carcinoma has been the cancer most consistently associated with body mass index (BMI) [2]. Each 5 kg/m² increase in BMI is linked to a 54% elevated risk of developing this cancer [3]. Genetic susceptibility for endometrial cancer include Lynch syndrome, which is caused by pathogenic germline variants in one of the mismatch repair (MMR) genes that maintain genomic stability. Lynch syndrome is the most common hereditary cause of endometrial cancer, and it is associated with 3% of all endometrial cancer cases [4, 5]. Other risk factors include diabetes [6], and hormonal-related factors, such as nulliparity [7, 8], postmenopausal estrogen-only hormone use [9], age at last birth [10], age at menarche [11], and oral contraceptive use [12]. Identifying other modifiable risk factors may help developing strategies to reduce the expected increasing incidence of these neoplasms.

Pesticides are a heterogeneous group of chemicals, including insecticides, fungicides and herbicides. These compounds are mainly used in agriculture for increasing food-production productivity and decreasing food-borne and vector-borne diseases [13]. Historically, cereals, olive trees, and vineyards dominated land use in Spain. In recent decades, intensive vegetable farming has grown significantly. Pesticide use has been led by insecticides and fungicides, followed by herbicides until the mid-70s of the last century [14]. In the last decades, European Union has been implementing measures on use and distribution of pesticides aimed to reduce environmental and health risks while maintaining crop productivity and improving controls [15]. Nevertheless, there has been controversy on the safety of certain pesticides as a consequence of diverging results from various assessments in their potential carcinogenicity [15, 16].

Oxidative stress, disruption of methyltransferases activity and epigenetic alterations are some mechanisms related to pesticide exposure that may lead to cancer development and other chronic diseases [16, 17]. Certain pesticides are also considered endocrine disruptors as certain compounds can interact with estrogenic and androgenic pathways [18, 19]. Pesticides residues are found in air, water and soil [20]; exposure can unintentionally occur through consuming foods or liquids with pesticide residues or occupationally, during their manufacture and manipulation [17, 20]. Few studies have assessed the possible relationship between exposure to pesticides and endometrial cancer with different assessment methods, such as measuring levels of pesticides in serum [21, 22] and adipose tissue [23], with no positive results. However, measurements of short half-life pesticide levels in biological samples may only indicate recent exposures, and those of long half-life pesticide levels or their derivatives may not accurately reflect the actual exposure to these substances [24, 25], which are relevant limitations when evaluating its association cancer and other diseases [26].

Assessing longer exposure periods through occupational exposures can help to overcome limitations of studies measuring pesticides levels in biological samples [26]. Several epidemiologic studies have evaluated the relationship between occupational exposure to pesticides and the risk of hematologic, bladder, breast, and prostate cancers [27, 28]. However, to the best of our knowledge, no previous epidemiological studies have specifically evaluated the association between occupational exposure to pesticides and endometrial cancer. In this study, we evaluated the association between occupational exposure to pesticides and endometrial cancer using a job-exposure matrix (JEM) in the Screenwide case-control study.

## Materials and methods

### Study design

The current study utilized data from the Screenwide study, a case-control study conducted in Spain [29]. Consecutive cases of endometrial cancer were recruited from 2017 to 2021, with no age limit restrictions, as well as hospital controls frequency matched to cases by age. Hospital controls comprised patients both with and without benign gynecologic conditions. Gynecologic benign conditions included endometriosis, fibroids, benign cysts, prolapse, and polyps. Hospital controls without gynecologic conditions were enrolled in the study during their preoperative anesthesia evaluations for surgical procedures related to ophthalmic, traumatologic, or other non-gynecologic diseases. The response rates were 89.6% among cases, 80.5% for controls with benign gynecological pathology, and 76.8% for asymptomatic women attending hospital for non-gynecological diseases. Participants were excluded from the study if they were pregnant, had given birth within the past 8 weeks, received chemotherapy or radiotherapy treatment in the preceding 6 months, or had communication difficulties that prevented them from understanding the informed consent or answering the questionnaire, such as not being fluent in Spanish or having an intellectual disability. In our study we considered the epidemiologic data collected for the 180 consecutive incident endometrial cancer cases, as well as 218 hospital controls; controls included 146 women with benign gynaecological pathology and 72 women attending hospital for non-gynaecological diseases. Occupational data was missing in 8 participants, yielding a sample size of 174 cases and 216 controls for the present analyses (Supplemental Fig. 1).

### Data collection and exposure assessment

Data were collected through structured epidemiological questionnaires administered by trained personnel in personal interviews [29]. The questionnaire included basic epidemiologic information such as demographic factors, tobacco consumption, lifetime occupational history (including jobs held for at least 1 year), coffee and tea consumption, physical activity, family history of cancer, anthropometric factors, reproductive factors and exogenous hormone use, sun exposure, sleeping habits, and chronotype information (individual preference for morning or evening activity). Each occupation was independently coded by two industrial hygienists according to the Spanish National Classification of Occupations (CNO-94), the Spanish version of the International Standard Classification of Occupations 1988 (ISCO-88). The coding process was carried out blinded to the case-control status of the participants. An agreement was reached by consensus when discrepancies occurred between the two coders. Workplace exposures were then evaluated through MatEmESp, a JEM developed in 2009 and designed for Spanish working conditions that covered the period 1996–2005 [30]. A JEM is a tool used to assess exposure to potential health hazards in occupational epidemiological studies. It comprises a list of levels of exposure to a variety of potentially harmful agents for selected occupational titles. In large population-based epidemiological studies, JEMs may be used as a quick and systematic means of converting coded occupational data (job titles) into a matrix of possible exposures, eliminating the need to assess each individual’s exposure in detail. The JEM exposure scores reflect the likelihood that a person’s exposure from their occupation is a significant contributor to their overall exposure compared to other sources. MatEmESp includes occupational exposure estimates in five categories based on job titles coded according to the CNO-94 coding system [30]: safety, ergonomics, hygiene, work conditions and psychosocial factors. Related to work conditions, identification of potentially exposed to pesticides occupations in MatEmESp was based on those occupations considered in the Finnish Job-Exposure Matrix (FINJEM) [31] and was extensively extended and adapted to Spanish working conditions by local experts [30]. Exposure to pesticide active compounds in the MatEmESp was based on use, toxicological relevance, legal status of the use in Spain, and existence of professional exposure limits. In particular, ten different active compounds were selected: four insecticides (endosulfan, methomyl, pyrethrin, and chlorpyrifos), four herbicides (2,4D, atrazine, diquat, and diuron), and two fungicides (captan and thiram) [32].

Supplemental Table 1 shows those job titles exposed to pesticides according to MatEmESp in Screenwide study. MatEmESp included quantitative indicators of probability (proportion of workers exposed by chemical and job title) and intensity of exposure (annual average environmental levels by chemical and job title). Duration, age at first exposure, time since first exposure and time since last exposure to pesticides were calculated based on the years at start and stop reported for each job and/or the date of interview. As MatEmESp covered the 1996–2005 period, similar exposures scores than those for the 1996–2005 period were assigned for exposures occurring outside the 1996–2005 timeframe. We adjusted the duration of exposure when participants reported holding multiple concurrent occupations that involved exposure to the same active substance simultaneously. This adjustment aimed to avoid overestimating the duration of exposure and was determined by the number of simultaneous jobs during the overlapping period by inversely weighting duration by the number of overlapping jobs during the corresponding period. Cumulative exposure scores (CES) were calculated for each compound as the result of the product of probability, intensity and duration (in years) of exposure. Continuous variables were categorized using median as the cut-off point based on the distribution among exposed controls. We grouped the job titles potentially exposed to pesticides into three different scenarios of exposure: (a) agricultural, poultry and livestock activities, (b) cleaning staff, (c) manufacturing and lumber industries. The latter group includes factory workers in the manufacture of pesticides and in wood production, as timber is often treated for pest control. Participants that reported no employment history (housewives, N = 28) were classified as never occupationally exposed to pesticides.

### Statistical analyses

The distribution of potential risk factors between cases and controls was compared using the Pearson’s chi-squared test. Calculated CES were normalized and calculated per 1 standard deviation (SD) increase. Correlation among exposed was calculated comparing each pesticide application group using Pearson’s correlation coefficients. Multivariate unconditional logistic regression models were used to estimate odds ratios (OR) and 95% confidence intervals (95% CI) for the association between occupational exposure to pesticides and endometrial cancer. The variables considered for inclusion in the multivariable models are shown in the Directed Acyclic Graph (Supplemental Fig. 2). Basic adjusted models included age at interview (< 60, 60–69, ≥ 70) and educational level (primary or less, secondary, higher). Variables for multivariate models were selected using the stepwise selection method, which in addition to basic adjustments, included body mass index (BMI; <25, 25–29.9 or ≥ 30), hormonal contraceptives use (ever, never) and menopause status (premenopause, postmenopause). The statistical significance level (alpha) was set at 0.05. For all variables, missing data was < 10% of subjects. Missing values were introduced in models as independent categories.

We conducted sensitivity analyses excluding housewives and stratified analyses by BMI and by type of control. Certain solvents have been previously associated to gynaecological tumours [21, 33]. Therefore, sensitivity analysis excluding participants who reported any occupational exposure to solvent compounds were performed to ensure that exposure to solvents was not influencing the association between pesticides and endometrial cancer. We assumed that earlier exposures were at least similar or higher than the ones estimated by MatEmESp in 1996–2005. However, exposures occurring after 2005 could be lower than estimated by the MatEmESp, as exposures are expected to decrease over time. Therefore, we performed sensitivity analyses excluding exposures after 2005, (4 registries from 2 cases and 2 controls).

We determined that, in order to estimate odds ratios of at least 2.3 with 80% power and assuming a 10% prevalence of exposure in controls, a sample size of 171 cases and 214 controls, maintaining a case-to-control ratio of 1.25, was required. Similarly, for odds ratios of at least 2.0 with the same power but assuming a 20% prevalence of exposure in controls, a sample size of 156 cases and 195 controls was deemed necessary. All analyses were conducted using R version 4.2.2.

### Ethical approval

The Screenwide study followed the national and international directives on ethics and data protection (Declaration of Helsinki and subsequent amendments; EU Reglament 2016/679) and the Spanish laws on data protection (Organic Law 3/2018; Law 14/2007 biomedical research). Participation in the study was voluntary, and all eligible subjects signed an informed consent form after receiving information about the study, before participating in any intervention. Study protocol was approved by the Ethics Committee for Clinical Research from the Bellvitge University Hospital.

## Results

### Demographic features of participants

Baseline demographic characteristics are shown in Table 1. The median age of cases was 67 (IQR 59–74), while controls had a median age of 66 (IQR 55–73). Cases were more likely to have a higher BMI (p-value < 0.001), to be diabetic (p-value = 0.014) and hypertense (p-value = 0.010). The proportion of postmenopausal participants was higher among cases than controls (p-value < 0.001). Controls were more likely to have used hormonal contraceptives (p-value = 0.003) compared to cases. Among the control group, there were no associations between occupationally exposed to pesticides and never exposed. (Table 2).

**Table 1:** Descriptive characteristics of the study population

	Controls No. (%)^a^	Cases No. (%)^a^	p-value^b^
Overall^c^	216 (55.4)	174 (44.6)	
Age			0.701
< 60	69 (31.9)	49 (28.2)	
60–69	68 (31.5)	56 (32.2)	
≥ 70	79 (36.6)	69 (39.7)	
Country of birth			0.165
Spain	199 (92.1)	153 (87.9)	
Other countries	17 (7.9)	21 (12.1)	
Educational level			0.382
Primary	163 (75.5)	126 (72.4)	
Secondary	37 (17.1)	28 (16.1)	
Higher	16 (7.4)	20 (11.5)	
BMI^d^			< 0.001
Underweight and normal < 25	64 (29.6)	26 (14.9)	
Overweight 25-29.9	81 (37.5)	50 (28.7)	
Obesity ≥ 30	63 (29.2)	92 (52.9)	
Diabetes			0.014
No	187 (86.6)	134 (77.0)	
Yes	29 (13.4)	40 (23.0)	
Hypertension			0.010
No	129 (59.7)	81 (46.6)	
Yes	87 (40.3)	93 (53.4)	
Hypercholesterolemia			0.290
No	127 (58.8)	93 (53.4)	
Yes	89 (41.2)	81 (46.6)	
Smoking			0.365
Never smoker	146 (67.6)	125 (71.8)	
Former smoker	70 (32.4)	49 (28.2)	
Occupational exposure to solvents			0.215
Never exposed	147 (68.1)	125 (71.8)	
Ever exposed	69 (31.9)	49 (28.2)	
Occupational shifts			0.172
Never night shift	175 (81.0)	150 (86.2)	
Ever night shift	41 (19.0)	24 (13.8)	
Family history of gynecological cancer^e^			0.513
Gynecological cancer	47 (21.8)	32 (18.4)	
Other type of cancer	91 (42.1)	69 (39.7)	
No gynecological cancer family history	76 (35.2)	70 (40.2)	
Age at menarche			0.543
< 13	94 (43.5)	81 (46.6)	
≥ 13	112 (51.9)	85 (48.9)	
Hormonal contraceptives			0.005
Never	98 (45.4)	104 (59.8)	
Ever	118 (54.6)	70 (40.2)	
Parity (number of children)			0.793
Nulliparous	29 (13.4)	24 (13.8)	
1	39 (18.1)	26 (14.9)	
2	95 (44.0)	76 (43.7)	
≥ 3	52 (24.1)	48 (27.6)	
Age at first delivery^f^			0.841
< 26	106 (57.0)	87 (58.0)	
≥ 26	79 (42.5)	62 (41.3)	
Menopause status			< 0.001
Premenopause	31 (14.4)	7 (4.0)	
Postmenopause	185 (85.6)	167 (96.0)	
Postmenopausal hormone therapy^g^			0.494
Never	164 (88.6)	147 (88.0)	
Ever	8 (4.3)	10 (6.0)	

No. = number, % = percentage

a Percentages do not sum to the total due to missing values

b Chi squared, calculated without missing values

c Row percentage, the rest of percentages in the table are column percentages

d Body mass index (BMI), expressed as weight (kg)/height2 (m2)

e Includes family history of endometrium, breast, ovary, uterus and/or uterine cancer

f Among parous women

g Among postmenopausal women

**Table 2:** Descriptive characteristics among controls by occupational exposure to pesticides

	Never exposed No. (%)^a^	Ever exposed No. (%)^a^	p-value^b^
Overall^c^	190 (88.0)	26 (12.0)	
Age			0.304
< 60	63 (33.2)	6 (23.1)	
60–69	61 (32.1)	7 (26.9)	
≥ 70	66 (34.7)	13 (50.0)	
Country of birth			0.971
Spain	175 (92.1)	24 (92.3)	
Other countries	15 (7.9)	2 (7.7)	
Educational level			0.183
Primary	140 (73.7)	23 (88.5)	
Secondary	34 (17.9)	3 (11.5)	
Higher	16 (8.4)	0 (0.0)	
BMI^d^			0.489
Underweight and normal < 25	55 (28.9)	9 (34.6)	
Overweight 25-29.9	70 (36.8)	11 (42.3)	
Obesity ≥ 30	58 (30.5)	5 (19.2)	
Diabetes			0.763
No	164 (86.3)	23 (88.5)	
Yes	26 (13.7)	3 (11.5)	
Hypertension			0.530
No	112 (58.9)	17 (65.4)	
Yes	78 (41.1)	9 (34.6)	
Hypercholesterolemia			0.467
No	110 (57.9)	17 (65.4)	
Yes	80 (42.1)	9 (34.6)	
Smoking			0.278
Never smoker	126 (66.3)	20 (76.9)	
Former smoker	64 (33.7)	6 (23.1)	
Occupational exposure to solvents			0.558
Never exposed	128 (67.4)	19 (73.1)	
Ever exposed	62 (32.6)	7 (26.9)	
Occupational shifts			0.102
Never night shift	157 (82.6)	18 (69.2)	
Ever night shift	33 (17.4)	8 (30.8)	
Family history of gynecological cancer^e^			0.383
Gynecological cancer	41 (21.6)	6 (23.1)	
Other type of cancer	83 (43.7)	8 (30.8)	
No gynecological cancer family history	64 (33.7)	12 (46.2)	
Age at menarche			0.861
< 13	83 (43.7)	11 (42.3)	
≥ 13	98 (51.6)	14 (53.8)	
Hormonal contraceptives			0.240
Never	89 (46.8)	9 (34.6)	
Ever	101 (53.2)	17 (65.4)	
Parity (number of children)			0.066
Nulliparous	28 (14.7)	1 (3.8)	
1	36 (18.9)	3 (11.5)	
2	85 (44.7)	10 (38.5)	
≥ 3	41 (21.6)	11 (42.3)	
Age at first delivery^f^			0.060
< 26	88 (54.3)	18 (75.0)	
≥ 26	73 (45.1)	6 (25.0)	
Menopause status			0.103
Premenopause	30 (15.8)	1 (3.8)	
Postmenopause	160 (84.2)	25 (96.2)	
Postmenopausal hormone therapy^g^			0.255
Never	141 (88.1)	23 (92.0)	
Ever	8 (5.0)	0 (0.0)	

No. = number, % = percentage

^a^ Percentages do not sum to the total due to missing values.

^b^ Chi squared, calculated without missing values.

^c^ Row percentage, the rest of percentages in the table are column percentages.

^d^ Body mass index (BMI), expressed as weight (kg)/height^2^ (m^2^).

^e^ Includes family history of endometrium, breast, ovary, uterus and/or uterine cancer.

^f^ Among parous women.

^g^ Among postmenopausal women.

### Associations between endometrial cancer and exposure to pesticides

Overall, 12% of controls and 18% of cases were occupationally exposed to pesticides (Table 3). Occupational exposure to pesticides was associated with endometrial cancer (OR = 2.08; 95% CI = 1.13–3.88 compared to non-exposed). We observed positive associations with earlier exposures in time. In particular, associations were observed for exposures that occurred before 2004 (OR = 2.35; 95% CI = 1.08–5.30). Similarly, associations were observed among those who started exposure at age 32 or more (OR = 2.69; 95% CI = 1.24–6.06), whose first exposure started ≥ 32 years or finished ≥ 13 years before the interview (OR = 2.39; 95% CI = 1.06–5.51 and OR = 2.35; 95% CI = 1.08–5.30, respectively). An exposure duration of less than 16 years was associated with endometrial cancer (OR = 2.43; 95% CI = 1.14–5.38), while an OR of 1.11 (95% CI = 0.38–3.04) was observed for a longer duration. Normalized cumulative exposure score showed no significant associations (OR = 1.25; 95% CI = 0.70–2.38).

**Table 3:** Associations between endometrial cancer and occupational exposure to pesticides

	Controls	Cases	OR (95% CI) ^a^
	N = 216	N = 174	
Never exposed to pesticides	190	142	Ref
Ever exposed to pesticides	26	32	2.08 (1.13–3.88)*
Ever exposed to insecticides	26	32	2.08 (1.13–3.88)*
Ever exposed to fungicides	6	17	4.40 (1.65–13.33)*
Ever exposed to herbicides	5	16	5.25 (1.84–17.67)*
Duration (years) ^b^			
< 16	13	22	2.43 (1.14–5.38)*
≥ 16	13	7	1.11 (0.38–3.04)
Age at first exposure ^b^			
< 32	13	21	2.69 (1.24–6.06)*
≥ 32	13	8	1.02 (0.37–2.67)
Years since first exposure ^b^			
< 32	13	11	1.34 (0.53–3.34)
≥ 32	13	18	2.39 (1.06–5.51)*
Years since last exposure ^b^			
< 13	13	9	1.29 (0.49–3.34)
≥ 13	13	20	2.35 (1.08–5.30)*
Year of last exposure ^b^			
< 2004	13	20	2.35 (1.08–5.30)*
≥ 2004	13	9	1.29 (0.49–3.34)
Normalized CES among exposed ^b, c^	26	29	1.21 (0.59–2.62)

OR = odds ratio, CI = confidence interval.

^*^ p-value < 0.05

^a^ Adjusted for age, educational level, BMI, hormonal contraceptives and menopausal status.

^b^ n in exposed cases do not sum to the total exposed cases due to missing values.

^c^ Per 1 standard deviation (SD) increase.

**Fig. 1:**
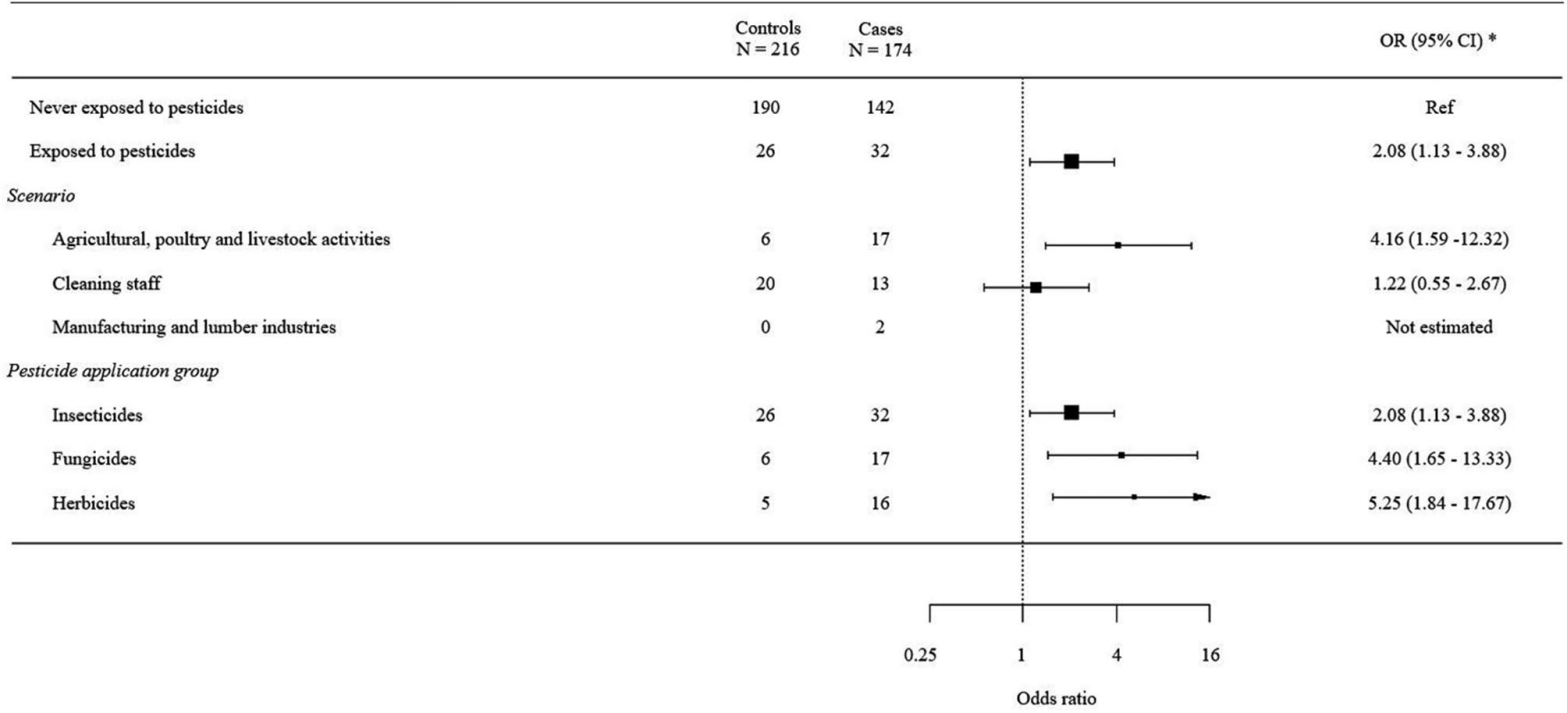
Forest plot of associations on pesticides by scenario and type of pesticide. * Adjusted for age, educational level, body mass index, hormonal contraceptives and menopausal status

Occupational exposure to each pesticide application group, including insecticides (OR = 2.08; 95% CI = 1.13–3.88), fungicides (OR = 4.40; 95% CI = 1.65–13.33) and herbicides (OR = 5.25; 95% CI = 1.84–17.67), were positively associated with endometrial cancer (Table 3; Fig. 1). However, independent effects for each pesticide application group were difficult to assess due to the correlation between exposures (Supplemental Table 2). Working as cleaning staff was not associated with endometrial cancer (OR = 1.22; 95% CI = 0.55–2.67). Contrarily, pesticide exposure related to agricultural, poultry and livestock activities revealed a positive association (OR = 4.16; 95% CI = 1.59–12.32).

### Sensitivity analyses

In general, analyses restricted to participants unexposed to solvents yielded similar patterns on the association between pesticide exposure and endometrial cancer (Supplemental Tables 3, OR_ever_ exposed = 2.44; 95% CI = 1.15–5.30). Similarly, excluding housewives from the analyses yielded similar patterns of associations (OR_ever_ exposed = 2.08, 95% CI = 1.12–3.91, data not shown). Stratified analyses by type of control yielded positive estimates among controls with benign gynaecologic pathology (Supplemental Tables 4, OR_gynaecologic_ = 2.08, 95% CI = 1.04–4.36; OR_non−gynaecological_ = 1.86, 95% CI = 0.83–4.44). Stratified analyses by BMI did not reveal clear patterns (Supplemental Table 5). Results were virtually identical excluding exposures occurring after 2005 (data not shown).

## Discussion

### Main findings

We observed a positive association between occupational exposure to pesticides and endometrial cancer using data from a recent case-control study in Spain. The three application groups (insecticides, fungicides and herbicides) were positively associated with endometrial cancer, although independent effects were difficult to assess due to correlation between exposures. Exposure to pesticides in the agricultural, poultry and livestock activities scenario was positively associated with endometrial cancer. On the contrary, the cleaning staff scenario did not reveal associations. These latter null results could be the result of a lower intensity and probability of exposure to pesticides compared with those in farmer and related occupations. Cumulative exposure scores did not show clear patterns, while exposures that occurred farther in the past were significantly associated with endometrial cancer. A short duration of the exposure was also associated with endometrial cancer, although further studies are required to untangle the relationship between the timing of exposure and its impact on endometrial cancer.

The results of our study suggest a positive association between occupational exposure to pesticides and endometrial cancer. Current evidence suggests that multiple mechanisms are involved in toxicity of pesticides [17, 28]. Pesticides can cause cellular and molecular alterations, such as oxidative stress, interference with methyltransferase activity and genotoxic effects, which may increase the risk of cancer [16, 17, 34]. Pesticides such as DDT (insecticide), glyphosate (herbicide) and mancozeb (fungicide) have recently been shown to have in vitro effects on endometrial cells [35–37]. Despite these disclosing insights on the potential carcinogenicity on endometrial tissue, the effects of environmental pesticide on human health have yet to be well defined. Additionally, some pesticides are considered endocrine disruptors due to their ability to interact with estrogenic and androgenic pathways, inhibit or induct aromatase activity, and disrupt the hypothalamic control of hormone levels, among other mechanisms [18]. The authors from a recent review suggested that there may be a potential association between exposure to endocrine disrupting chemicals and endometrial cancer, but the specific molecular pathways are yet unclear [19]. Factors such as frequency of exposure, specific types of pesticides, their metabolites and persistence in the organism could play a role in the potential carcinogenic effect of pesticides [19, 27, 38].

### Previous results

Assessing the association between pesticide exposure and long-latency diseases is still a challenge due to the complexity of the pathways involved and limitations in exposure assessments [37, 38]. Only three studies have evaluated pesticides and endometrial cancer with heterogenous methodologies [21–23]. In particular, pesticides levels were evaluated in serum [21, 22] and adipose tissue [23] and yielded negative associations. However, two of them had limited sample sizes (below 100 cases and/or below 40 controls) [22, 23]. In addition, they evaluated compounds with long half-lives, such as DDT. However, little is known regarding the rest of pesticides with shorter half-lives, such as glyphosate [24], and evaluating its levels in biosamples may reflect recent exposures rather than long-term exposure to these compounds [25]. In this regard, occupational exposure assessments using JEMs have been proposed in evaluating lifelong exposure in population-based studies of diseases with long-latency periods, such as cancer [39].

Pesticide exposure in the agricultural activities scenario was positively associated with endometrial cancer in this study. In the last decade, many studies have assessed risk of cancers other than endometrial and exposure to pesticides among these workers [27, 28]. There is increasing evidence that occupational pesticide exposure influences the risk of certain cancers in agricultural workers [27], although with inconsistencies as some studies suffer from confounding [28, 40, 41]. Participants in our study who had exposure to pesticides farther in the past showed associations with endometrial cancer. The changing regulations of these compounds and the increasing use of personal protective equipment over time may explain these associations. Nowadays, many farm workers and employers overlook the importance of personal protective equipment and adequate pesticide handling training [42]. Thus, future investigations should consider the possible uneven use of personal protective equipment to accurately estimate pesticide exposure in both agricultural and other occupational settings.

### Strengths and limitations

To the best of our knowledge, our study is the first to assess the potential association between occupational exposure to pesticides and endometrial cancer. We counted with detailed data that allowed us to potentially control for confounding for several factors. We did not observe clear evidence of confounding, although residual confounding cannot be completely discarded in explaining some of our results. In addition, those working in agriculture scenario might be exposed to other common exposures beyond pesticides, including infectious agents, that could potentially act as confounding factors in the observed association. We used a JEM to assess pesticides exposure, which may overcome previous exposure assessment limitations, and JEMs perform better than self-reported occupational exposures [43]. They represent an efficient method to estimate a wide range of exposures, although they can lead to substantial exposure misclassification [44]. Dosemeci et al., showed that several strategies improved JEM exposure assessment, which included considering the industry sectors of economic activities involved in specific occupations, accounting for differences in exposures over time considering periods of predominant use; and including both intensity and frequency of exposures in the assessments. Our JEM considers intensity and frequency of exposures, but it does not consider the industry sectors of economic activities, nor does it account for differences in exposures over time. In addition, a different use of personal protective equipment may occur within a same occupation, which can also contribute to exposure misclassification [45]. However, as exposure assessment was blind to the case-control status, misclassification would result in the attenuation for exposure estimates [46], which would reinforce our conclusions. The JEM exposure scores reflect the likelihood that a person’s exposure from their occupation is a significant contributor to their overall exposure compared to other sources. In this regard, we could not assess non-occupational potential sources of exposure to pesticides, including dietary factors, residential pesticide use, personal care or household cleaning products. Combining both direct and indirect exposure assessment methods will facilitate a thorough evaluation of occupational exposures and reduce the likelihood of exposure misclassification. Additionally, controls with benign gynaecological conditions were also included, which could lead to selection bias. Controls with benign gynaecological conditions may have a different distribution of risk factors compared to the general population, particularly with regards to hormonal exposures. However, the estimates were also almost two-fold excluding these controls, suggesting that this bias may not drive the associations. The lower response rate of controls compared with cases might have introduced selection bias. Controls of lower socioeconomic level may be less likely to participate, and socioeconomic level may be associated to exposure to pesticides. However, we did not observe significant differences in education level between cases and controls. This is the largest study on pesticides and endometrial cancer. However, our results should be cautiously interpreted given that sample size was limited for subgroup analyses, and the scenario that showed a greater risk (agriculture) was small. Additional evaluations are needed to confirm this association and to assess the impact of the various compounds and exposure scenarios.

### Conclusions

Assessment of occupational exposure to pesticides assessed using a Spanish JEM revealed a positive association with endometrial cancer. Additional large population-based studies and detailed exposure assessments are needed to confirm our results. The elucidation of the role of pesticide compounds on endometrial cancer should shed a light on the aetiology of this tumour and help the implementation of appropriate public health policies to mitigate its expected increasing burden.

### Electronic supplementary material

Below is the link to the electronic supplementary material.

Supplementary Material 1: Table S1: Occupations considered exposed to pesticides in the Screenwide case-control study. Table S2: Pearson’s correlation coefficients between cumulative exposure scores (CES) for pesticides and for each pesticide application group, among exposed to pesticides.; Table S3: Associations between endometrial cancer and pesticide exposure, among the unexposed to solvents. Table S4: Associations between endometrial cancer and pesticide exposure, by type of control.; Table S5: Associations between endometrial cancer and pesticide exposure, by BMI; Figure S1: Flow chart; Figure S2: Directed acyclic graph (DAG).

## Data Availability

Data are available from the corresponding author at lcostas@iconcologia.net on reasonable request.
