# Supplementary material for: Occupational exposure to pesticides and endometrial cancer in the Screenwide case-control study
Source: Environ Health. 2023 Nov 2;22:77. doi: 10.1186/s12940-023-01028-0 (PMC10621144; doi:10.1186/s12940-023-01028-0)

**Supplementary material**

[Supplemental table 1 2](#_Toc145878518)

[Supplemental table 2 3](#_Toc145878519)

[Supplemental table 3 4](#_Toc145878520)

[Supplemental table 4 5](#_Toc145878521)

[Supplemental table 5 6](#_Toc145878522)

[Supplemental figure 1 7](#_Toc145878523)

[Supplemental figure 2 8](#_Toc145878524)

# Supplemental table 1

Occupations considered exposed to pesticides in the Screenwide case-control study.

| **CNO-94^a^** | **ISCO-88^b^** | **Description^c^** | **Screenwide study (n) ^d^** |
| --- | --- | --- | --- |
| 3122 | 3227 | Veterinary assistants | 1 |
| 6111 | 6121 | Dairy and livestock producers | 1 |
| 8141 | 8141 | Wood-processing-plant operators | 1 |
| 9121 | 9132 | Helpers and cleaners in offices, hotels and other establishments | 33 |
| 9410 | 9211 | Farmhands and labourers | 4 |
| 6011 | 6111 | Field crop and vegetable growers | 15 |
| 6112 | 6122 | Poultry producers | 2 |
| 9700 | 932 | Manufacturing labourers | 1 |

a Spanish classification of occupations (1994).

b International Standard Classification of Occupations (1988).

c Description based on ISCO-88.

d Job available in Screenwide case-control study.

# Supplemental table 2

Concordance and correlation coefficients between exposures among controls. Cohen's Kappa coefficients are computed for binary variables (ever/never). Correlation coefficients are computed for duration of exposures in years.

|  | Pesticides | | Insecticides | | Fungicides | |
| --- | --- | --- | --- | --- | --- | --- |
|  | Kappa | Correlation | Kappa | Correlation | Kappa | Correlation |
| Insecticides | 1.000 | 1.000 |  |  |  |  |
| Fungicides | 0.345 | 0.925 | 0.345 | 0.925 |  |  |
| Herbicides | 0.295 | 0.977 | 0.295 | 0.977 | 0.907 | 1.000 |

# Supplemental table 3

Associations between endometrial cancer and pesticide exposure, among the unexposed to solvents.

|  | Controls,  N = 147 | Cases,  N = 125 | OR (95% CI) ^a^ |  |
| --- | --- | --- | --- | --- |
| Never exposed to any pesticide | 128 | 102 | Ref |  |
| Ever exposed to pesticides | 19 | 23 | 2.44 (1.15-5.30)* |  |
| Cumulative exposure score (CES) ^b^ |  |  |  |  |
| Below median | 8 | 6 | 1.67 (0.47-5.80) |  |
| Above median | 11 | 15 | 2.45 (0.99-6.27) |  |
| Scenario |  |  |  |  |
| Agricultural, poultry and livestock activities | 5 | 14 | 4.59 (1.56-15.70)* |  |
| Cleaning staff | 14 | 8 | 1.41 (0.50-3.81) |  |
| Manufacturing and lumber industries | 0 | 1 | Not estimated |  |
| Pesticide application group |  |  |  |  |
| Insecticides | 19 | 23 | 2.44 (1.15-5.30)* |  |
| Fungicides | 5 | 15 | 5.27 (1.77-18.18)* |  |
| Herbicides | 5 | 15 | 5.27 (1.77-18.18)* |  |
| Duration (years) ^b^ |  |  |  |  |
| <16 | 9 | 15 | 2.89 (1.13-7.83)* |  |
| ≥16 | 10 | 6 | 1.36 (0.41-4.34) |  |
| Age at first exposure ^b^ |  |  |  |  |
| <32 | 8 | 18 | 4.41 (1.71-12.40)* |  |
| ≥32 | 11 | 3 | 0.54 (0.11-2.00) |  |
| Years since first exposure ^b^ |  |  |  |  |
| <32 | 8 | 6 | 1.35 (0.38-4.60) |  |
| ≥32 | 11 | 15 | 2.78 (1.11-7.27)* |  |
| Years since last exposure ^b^ |  |  |  |  |
| <13 | 10 | 5 | 1.05 (0.29-3.42) |  |
| ≥13 | 9 | 16 | 3.39 (1.31-9.35)* |  |
| Year of last exposure ^b^ |  |  |  |  |
| < 2004 | 9 | 16 | 3.39 (1.31-9.35)* |  |
| ≥ 2004 | 10 | 5 | 1.05 (0.29-3.42) |  |
| OR = odds ratio, CI = confidence interval.  a Adjusted for age, educational level, BMI, hormonal contraceptives and menopausal status.  b n in exposed cases do not sum to the total exposed cases due to missing values. | | | | |

# Supplemental table 4

Associations between endometrial cancer and pesticide exposure, by type of control.

|  | Gynecologic controls,  N = 146 | | Cases,  N = 174 | OR (95% CI) ^a^ |  | Non-gynecologic controls,  N = 70 | Cases,  N = 174 | OR (95% CI) ^a^ |  |
| --- | --- | --- | --- | --- | --- | --- | --- | --- | --- |
| Never exposed to any pesticide | 132 | | 142 | Ref |  | 58 | 142 | Ref |  |
| Ever exposed to pesticides | 14 | | 32 | 2.08  (1.04-4.36)* |  | 12 | 32 | 1.86  (0.83-4.44) |  |
| Cumulative exposure score (CES) ^b^ |  |  |  |  |  |  |  |  |  |
| Below median | 7 | | 13 | 1.60  (0.60-4.62) |  | 5 | 13 | 1.84  (0.55-7.52) |  |
| Above median | 7 | | 16 | 2.11  (0.82-5.97) |  | 7 | 16 | 1.51  (0.56-4.47) |  |
| Scenario |  | |  |  |  |  |  |  |  |
| Agricultural, poultry and livestock activities | 4 | | 17 | 3.74  (1.28-13.75)* |  | 2 | 17 | 4.92  (1.25-33.05)* |  |
| Cleaning staff | 10 | | 13 | 1.26  (0.52-3.18) |  | 10 | 13 | 1.00  (0.37-2.82) |  |
| Manufacturing and lumber industries | 0 | | 2 | Not estimated |  | 0 | 2 | Not estimated |  |
| Pesticide application group |  | |  |  |  |  |  |  |  |
| Insecticides | 14 | | 32 | 2.08  (1.04-4.36)* |  | 12 | 32 | 1.86  (0.83-4.44) |  |
| Fungicides | 3 | | 17 | 4.76  (1.48-21.36)* |  | 3 | 17 | 3.42  (1.00-15.94) |  |
| Herbicides | 2 | | 16 | 7.04  (1.85-46.33)* |  | 3 | 16 | 3.26  (0.94 15.33) |  |
| Duration (years) ^b^ |  | |  |  |  |  |  |  |  |
| <16 | 8 | | 22 | 2.30  (0.99-5.89) |  | 5 | 22 | 2.54  (0.89-8.62) |  |
| ≥16 | 6 | | 7 | 1.19  (0.36-4.02) |  | 7 | 7 | 0.86  (0.25-3.07) |  |
| Age at first exposure ^b^ |  | |  |  |  |  |  |  |  |
| <32 | 9 | | 21 | 2.15  (0.93-5.34) |  | 4 | 21 | 3.64  (1.21-13.78)* |  |
| ≥32 | 5 | | 8 | 1.35  (0.42-4.76) |  | 8 | 8 | 0.55  (0.16-1.89) |  |
| Years since first exposure ^b^ |  | |  |  |  |  |  |  |  |
| <32 | 7 | | 11 | 1.26  (0.45-3.76) |  | 6 | 11 | 1.23  (0.37-4.62) |  |
| ≥32 | 7 | | 18 | 2.48  (0.98-6.91) |  | 6 | 18 | 1.97  (0.71-6.19) |  |
| Years since last exposure ^b^ |  | |  |  |  |  |  |  |  |
| <13 | 5 | | 9 | 1.56  (0.50-5.46) |  | 8 | 9 | 0.78  (0.24-2.58) |  |
| ≥13 | 9 | | 20 | 2.02  (0.87-5.05) |  | 4 | 20 | 3.13  (1.00-12.02) |  |
| Year of last exposure ^b^ |  | |  |  |  |  |  |  |  |
| < 2004 | 9 | | 20 | 2.02  (0.87-5.05) |  | 4 | 20 | 3.13  (1.00-12.02) |  |
| ≥ 2004 | 5 | | 9 | 1.56  (0.50-5.46) |  | 8 | 9 | 0.78  (0.24-2.58) |  |
| OR = odds ratio, CI = confidence interval.  ^a^ Adjusted for age, educational level, BMI, hormonal contraceptives and menopausal status.  ^b^ n in exposed cases do not sum to the total exposed cases due to missing values. | | | | | | | | |  |

# Supplemental table 5

Associations between endometrial cancer and pesticide exposure, by BMI.

|  |  |  | BMI <30 |  |  |  | BMI ≥30 |  |
| --- | --- | --- | --- | --- | --- | --- | --- | --- |
|  | Controls,  N = 145 | Cases,  N = 76 | OR (95% CI) ^a^ |  | Controls,  N = 63 | Cases,  N = 92 | OR (95% CI) ^a^ |  |
| Never exposed to any pesticide | 125 | 60 | Ref |  | 58 | 77 | Ref |  |
| Ever exposed to pesticides | 20 | 16 | 1.84  (0.84-4.01) |  | 5 | 15 | 2.32  (0.83-7.61) |  |
| Cumulative exposure score (CES) ^b^ |  |  |  |  |  |  |  |  |
| Below median | 10 | 5 | 1.13  (0.32-3.54) |  | 1 | 7 | 5.22  (0.86-100.55) |  |
| Above median | 10 | 9 | 2.11  (0.76-5.80) |  | 4 | 7 | 1.33  (0.37-5.46) |  |
| Scenario |  |  |  |  |  |  |  |  |
| Agricultural, poultry and livestock activities | 4 | 9 | 5.24  (1.55-20.88)* |  | 2 | 8 | 2.94  (0.68-20.38) |  |
| Cleaning staff | 16 | 7 | 1.03  (0.37- 2.65) |  | 3 | 5 | 1.31  (0.29-6.85) |  |
| Manufacturing and lumber industries | 0 | 0 | Not estimated |  | 0 | 2 | Not estimated |  |
| Pesticide application group |  |  |  |  |  |  |  |  |
| Insecticides | 20 | 16 | 1.84  (0.84-4.01) |  | 5 | 15 | 2.32  (0.83-7.61) |  |
| Fungicides | 3 | 8 | 6.80  (1.76-33.82)* |  | 3 | 9 | 2.36  (0.65-11.23) |  |
| Herbicides | 3 | 8 | 6.80  (1.76-33.82)* |  | 2 | 8 | 2.97  (0.68-20.64) |  |
| OR = odds ratio, CI = confidence interval.  ^a^ Adjusted for age, educational level, hormonal contraceptives and menopausal status.  ^b^ n in exposed cases do not sum to the total exposed cases due to missing values. | | | | | | | |  |

# Supplemental figure 1

Flow-chart of the selection of study population.


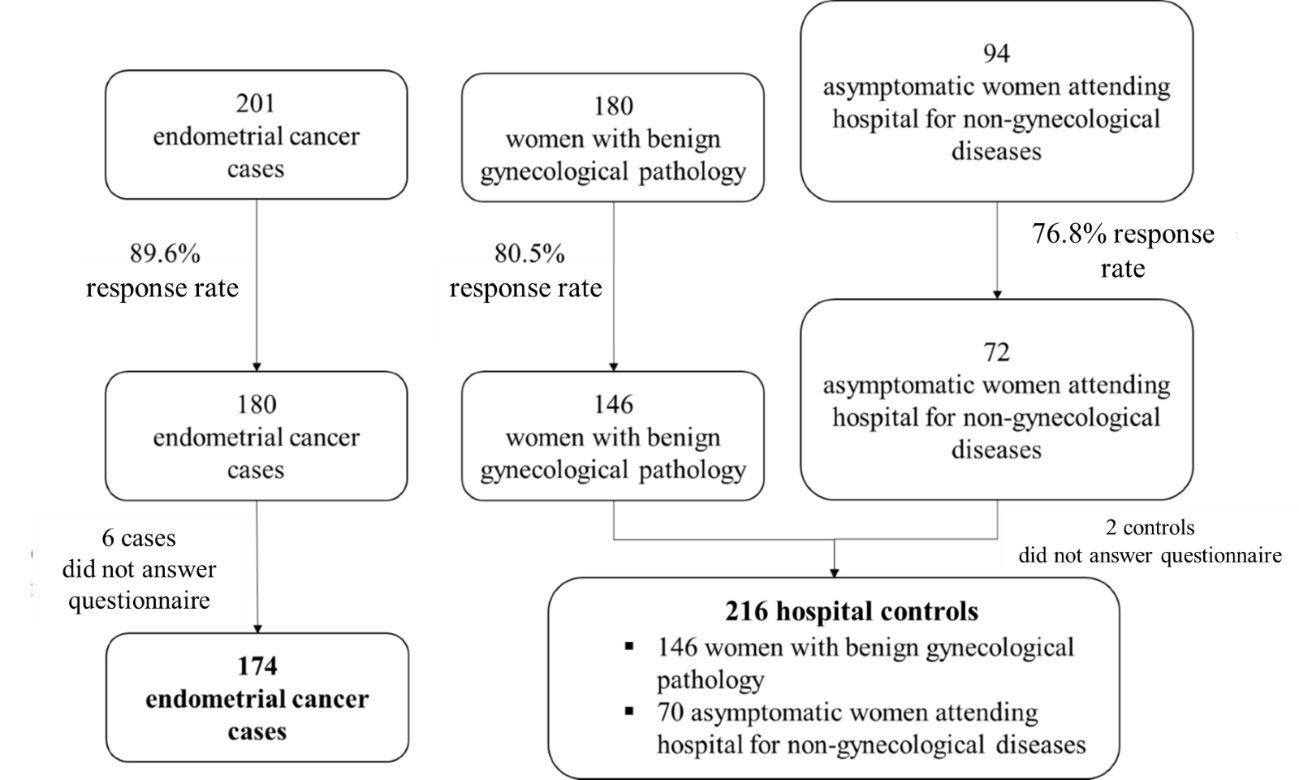


# Supplemental figure 2

Directed acyclic graph (DAG).

Variables significant in the descriptive analysis were included in the DAG. Age was also considered due to the matching between cases and controls, as well as educational level, which could have influenced the reported occupation.


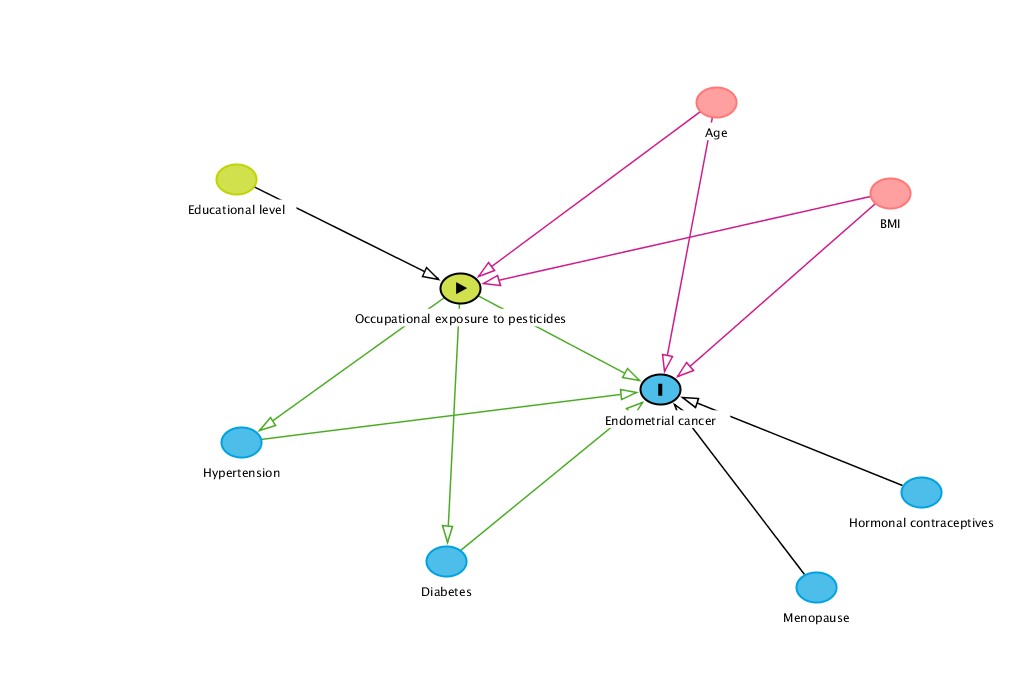

Supplement: Supplementary file 1 — Supplementary Material 1: Table S1: Occupations considered exposed to pesticides in the Screenwide case-control study. Table S2: Pearson’s correlation coefficients between cumulative exposure scores (CES) for pesticides and for each pesticide application group, among exposed to pesticides.; Table S3: Associations between endometrial cancer and pesticide exposure, among the unexposed to solvents. Table S4: Associations between endometrial cancer and pesticide exposure, by type of control.; Table S5: Associations between endometrial cancer and pesticide exposure, by BMI; Figure S1: Flow chart; Figure S2: Directed acyclic graph (DAG). [file 12940_2023_1028_MOESM1_ESM.docx]
